# A novel necroptosis-related lncRNA based signature predicts prognosis and response to treatment in cervical cancer

**DOI:** 10.3389/fgene.2022.938250

**Published:** 2022-12-06

**Authors:** Xinyi Du, Xiaowen Pu, Xintao Wang, Yuchen Zhang, Ting Jiang, Yanjun Ge, Haiyan Zhu

**Affiliations:** Department of Gynecology, Shanghai First Maternity and Infant Hospital, Tongji University School of Medicine, Shanghai, China

**Keywords:** necroptosis-related lncRNA, cervical cancer, immune, prognostic model, risk score

## Abstract

**Background:** Necroptosis has been demonstrated to play a crucial role in the prognosis prediction and assessment of treatment outcome in cancers, including cervical cancer. The purpose of this study was to explore the potential prognostic value of necroptosis-related lncRNAs and their relationship with immune microenvironment and response to treatment in cervical cancer.

**Methods:** Data from The Cancer Genome Atlas (TCGA) were collected to obtain synthetic data matrices. Necroptosis-related lncRNAs were identified by Pearson Correlation analysis. Univariate Cox and multivariate Cox regression analysis and Lasso regression were used to construct a necroptosis-related LncRNAs signature. Kaplan-Meier analysis, univariate and multivariate Cox regression analyses, receiver operating characteristic (ROC) curve, nomogram, and calibration curves analysis were performed to validate this signature. Gene set enrichment analyses (GSEA), immunoassays, and the half-maximal inhibitory concentration (IC50) were also analyzed.

**Results:** Initially, 119 necroptosis-related lncRNAs were identified based on necroptosis-related genes and differentially expressed lncRNAs between normal and cervical cancer samples. Then, a prognostic risk signature consisting of five necroptosis-related lncRNAs (DDN-AS1, DLEU1, RGS5, RUSC1-AS1, TMPO-AS1) was established by Cox regression analysis, and LASSO regression techniques. Based on this signature, patients with cervical cancer were classified into a low- or high-risk group. Cox regression confirmed this signature as an independent prognostic predictor with an AUC value of 0.789 for predicting 1-year OS. A nomogram including signature, age, and TNM stage grade was then established, and showed an AUC of 0.82 for predicting 1-year OS. Moreover, GSEA analysis showed that immune-related pathways were enriched in the low-risk group; immunoassays showed that most immune cells, ESTIMAT scores and immune scores were negatively correlated with risk score and that the expression of immune checkpoint-proteins (CD27, CD48, CD200, and TNFRSF14) were higher in the low-risk group. In addition, patients in the low-risk group were more sensitive to Rucaparib, Navitoclax and Crizotinib than those in the high-risk group.

**Conclusion:** We established a novel necroptosis-related lncRNA based signature to predict prognosis, tumor microenvironment and response to treatment in cervical cancer. Our study provides clues to tailor prognosis prediction and individualized immunization/targeted therapy strategies.

## 1 Introduction

Despite a downward trend in cervical cancer incidence due to the invention of the human papillomavirus (HPV) vaccine and widespread cervical cancer screening, cervical cancer (CC) remains the fourth most common malignant disease among women, with an estimated 604,000 new cases and 342,000 deaths worldwide in 2020 (Sung et al., 2021). High-risk HPV infection is regarded as a major cause of cervical cancer. Nevertheless, in addition to persistent HPV infection, the development of cervical cancer requires synergistic cancer-promoting factors (Cohen et al., 2019).

Necroptosis, a form of programmed necrotic cell death, has recently been reported to play a pivotal role in oncogenesis, cancer metastasis, and cancer immunity (Yan et al., 2022). In cervical cancer, RETRA (REactivation of Transcriptional Reporter Activity) induces necroptosis and increases ROS production (Mohanty et al., 2022), while receptor-interacting protein kinase 3 (RIPK3) expression is necessary for PolyIC-induced necroptosis (Schmidt et al., 2015). In addition, the hyponecrotic process may predict poor prognosis in HPV-positive cervical cancer by reducing tumor-associated M1 polarization of (Li et al., 2018).

Long non-coding RNAs (lncRNAs), a class of transcripts more than 200 nucleotides in length, have been suggested to play a pivotal role in tumor cell activity, participate in multi-gene regulatory networks, and serve as biomarkers for early tumor detection and prognosis (Huarte, 2015), including cervical cancer. LINC01305 suppresses the malignant progression of cervical cancer *via* miR-129-5p/Sox4 axis (Xu et al., 2020). HAND2-AS1 delays the progression of cervical cancer *via* its regulation of the microRNA-21-5p/TIMP3/VEGFA axis (Gao et al., 2021). Recent studies have detected a close correlation between lncRNAs and necroptosis (Jiang et al., 2021). NRF (necrosis-related factor) regulates programmed necrosis and myocardial injury during ischemia-reperfusion by targeting microRNA-873 (Wang et al., 2016), and lncRNA H19-derived microRNA-675 promotes liver necroptosis by targeting FADD (Harari-Steinfeld et al., 2021).

In recent years, multiple models have been developed to explore tumor mechanisms, assess cancer prognosis, and guide clinical treatment (Ghosh et al., 2017; Khajanchi and Nieto, 2021; Sardar et al., 2021). More recently, necroptosis-related lncRNAs have been extensively explored in predicting prognosis and immunotherapy response in gastric cancer (Zhao et al., 2021), stomach adenocarcinoma (Luo et al., 2022), breast cancer (Chen et al., 2022), lung adenocarcinoma (Lu et al., 2022), colon cancer (Liu et al., 2022). However, the potential role of necroptosis-related lncRNAs in cervical cancer remains to be clearly elucidated.

In the current study, we first analyzed necroptosis-related lncRNAs in cervical cancer, then constructed a novel signature based on necroptosis-related lncRNA, and finally investigated the prognostic value of the signature and its relationship with the immune microenvironment and treatment response in cervical cancer. Hoping that provides clues to tailor prognosis prediction and targeted therapy strategies.

## 2 Materials and methods

### 2.1 Obtaining information of patients with cervical cancer

The RNA transcriptome datasets (HTSeq-Counts and HTSeq-FPKM) and clinical information were downloaded from the Cancer Genome Atlas (TCGA) (https://portal.gdc.cancer.gov/) to obtain synthetic data matrices on cervical cancer, and then we used “data.table”, “tibble”, “dplyr” and “tidyr” packages to convert FPKM values into TPM values of the synthetic matrix. Thus, we obtained two synthetic data matrices. The Counts value matrix was used to identify differentially expressed lncRNAs, while the TPM value matrix was used for the other analyses. To reduce bias in this analysis, we excluded cervical cancer patients with missing overall survival (OS) values or short OS values (<30 days). Based on relevant clinical information, we retrieved 306 patients and randomized them into training group and testing group in a 7:3 ratio using the Strawberry Perl and caret R package.

### 2.2 Processing necroptosis-related genes and LncRNAs

After searching for the term “necroptosis” in MSigDB (http://www.gsea-msigdb.org/gsea/msigdb/index. jsp) and downloading necroptosis-related genes, using the necroptosis-related genes collated by Zhao et al. (2021), we obtained 67 profiles of necroptosis-related genes. Then, the synthesized data matrix was screened by the “limma”, “edgR” software package, and we identified 5022 differentially expressed lncRNAs (|Log2 fold change (FC)| > 1, false discovery rate (FDR) < 0.05, *p* < 0.05). Pearson correlation analysis was performed between 67 necroptosis-related genes and differentially expressed lncRNAs in the comprehensive matrices (|Pearson correlation coefficients| >0.3, and *p* < 0.001). Finally, 119 necroptosis-related lncRNAs were identified (Burk et al., 2017).

### 2.3 Construction and validation of the risk signature

Univariate Cox (uni-Cox), multivariate Cox (multi-Cox) regression analysis and lasso regression were used to identify necroptosis-related lncRNAs associated with OS based on clinical data of cervical cancer cases in TCGA (*p* < 0.05) (Qin et al., 2021). The risk score was calculated as follows (Wang et al., 2018):

$$risk\;score=\overset{n}{\underset{k=1}{\sum }}\left(\exp{lncRNA}^{k}*coef{lncRNA}^{k}\right)$$

Cervical cancer patients included in the study were divided into high- and low-risk groups based on the median risk score. The different OS times of the high- and low-risk groups were analyzed with the Kaplan—Meier plotter using the R package “survival”, “survminer”, “glmnet”, “caret”, “timeROC”, and “finalfit”. Then, the model was built. Time-dependent receiver operating characteristic (ROC) curves for the model were plotted at 1, 3 and 5 years by a calculation procedure.

### 2.4 Identification of independent prognostic factors and ROCs

Univariate Cox (uni-Cox) and multivariate Cox (multi-Cox) regression analyses were used to assess whether the signature and clinical characteristics were independent variable factors. Time-dependent ROC was used to evaluate the sensitivity and specificity of independent prognostic factors.

### 2.5 Nomogram and calibration

Nomograms for 1-year, 3-year, and 5-year OS were created using the “rms”, “regplot”, and “survivor” packages, and calibration curves based on the Hosmer-Lemeshow test were used to show whether the predicted results matched the actual results.

### 2.6 Gene set enrichment analyses

Curated gene sets (kegg.v7.4. symbols.gmt) were obtained from MSigDB (www.gsea-msigdb.org) and then the “clusterProfiler” package was used to identify significantly enriched pathways between the low- and high-risk groups (*p* < 0.05).

### 2.7 Immune cell infiltration analysis

Based on the GSEA results, we decided to analyze the immune-cell factors in the risk group. Immune cell infiltration was calculated on TIMER2.0 (http://timer.cistrome.org/) for TCGA cervical cancer samples, including TIMER, CIBERSORT, XCELL, QUANTISEQ, MCPcounter, EPIC, and CIBERSORT algorithm. In another way, we can download infiltration estimation profiles of all TCGA tumors on the same website. R packge estimate was used to estimate the level of stromal cells present and the level of immune cell infiltration in the tumor tissues based on expression data. Wilcoxon signed-rank test, “ggplot2” packages were performed in the analysis of the differences in the content of immune infiltrating cells, and the results were displayed in a bubble chart.

### 2.8 Exploration of drug therapy targeting risk model

To determine whether this signature could be used for drug therapy in cervical cancer patients, we calculated the half-maximal inhibitory concentration (IC50) of targeted agents on the cervical cancer patients’ dataset, dataset by “oncoPredict”. Commonly targeted agents for cancer include Rucaparib, AZD8055, Bryostatin1, BX795, CHIR-99021, veliparib, Palbociclib, Embelin, DMOG, and Crizotinib. The difference in IC50 between the two risk groups was compared by Wilcoxon’s test. Results were plotted with R packages “ggplot2”.

## 3 Results

### 3.1 Identification of necroptosis-related LncRNAs in cervical cancer patients

**FIGURE 1:**
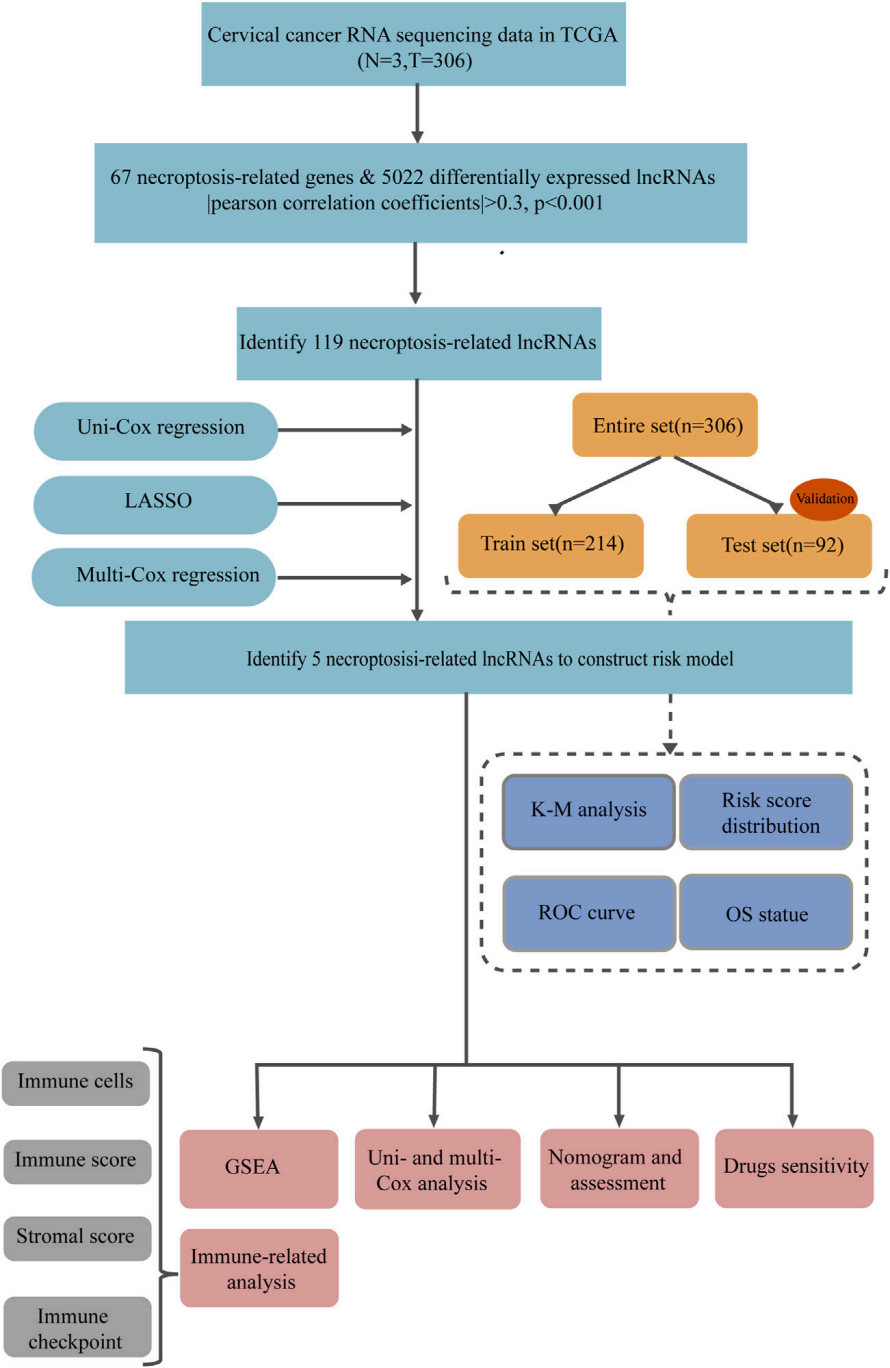
The flow chart.

The flow of the study was exhibited in Figure 1. Initially, we utilized gene expression and clinical information from three normal samples and 306 cancer samples to identify necroptosis-related lncRNAs in cervical cancer patients. According to the differentially expression of 67 necroptosis-related genes and lncRNAs between normal and tumor samples, we obtained 119 necroptosis-related lncRNAs (|correlation coefficients|> 0.3 and *p* < 0.001), of which we screened 52 upregulated lncRNAs and 67 downregulated lncRNAs with |fold change| > 1, adjusted *p* < 0.05. Differentially expressed lncRNAs in normal and tumor tissues and necroptosis-related lncRNAs were visualized by heatmap and volcano plot using the “ggplot2” and “pheatmap” packages of R software in Supplementary Figures S1–S2.

### 3.2 Construction of a novel signature based on necroptosis-related LncRNAs

**FIGURE 2:**
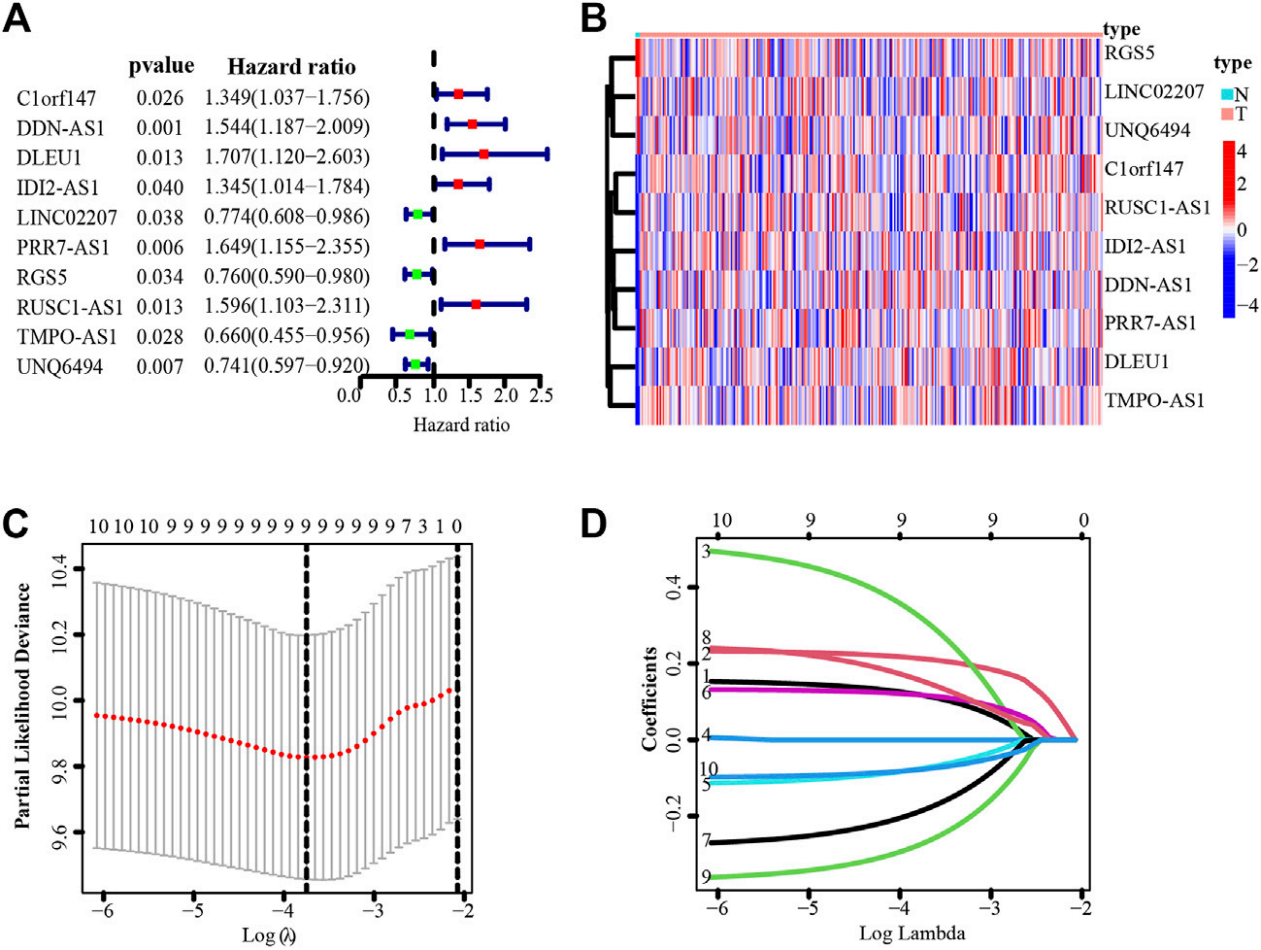
**I**dentification of necroptosis-related lncRNAs with prognostic value in cervical cancer patients. **(A)** Forest plot showed the prognostic risk value of the 10 necroptosis-related lncRNAs *via* univariate Cox regression analysis. **(B)** Heatmap of 10 necroptosis-related lncRNAs. **(C)** Cross-validation for optimizing the parameter in LASSO regression. **(D)** The influence of log lambda on the weight coefficients of each independent variable.

Next, we explored the prognostic potential of necroptosis-related lncRNAs in cervical cancer patients. Using univariate Cox regression analysis, we detected 10 necroptosis-related lncRNAs significantly correlated with overall survival (OS) (all *p* < 0.05) (Figures 2A,B). Based on the 10 necroptosis-related lncRNAs screened above, further randomized LASSO regression technique was performed to construct signatures, and the results showed that nine necroptosis-related lncRNAs were appropriate for constructing prognostic signatures (Figures 2C,D) (Supplementary Table S1). Then we obtained five necroptosis-related lncRNAs by multivariate Cox regression analysis, of which 3 lncRNAs were upregulated and two IncRNAs were downregulated (Table 1). We then calculated the risk score using the following formula:

$$\begin{array}{l} risk\;score=DDN-AS1\times \left(0.2805\right)+DLEU1\times \left(0.5993\right)\\ +RGS5\times \left(-0.3592\right)+RUSC1-AS1\times \left(0.4950\right)\\ +TMPO-AS1\times \left(-0.4184\right) \end{array}$$

**TABLE 1:** The coefficients (coef) of the five necrosis-related lncRNAs with prognostic significance.

LncRNAs	DDN-AS1	DLEU1	RGS5	RUSC1-AS1	TMPO-AS1
Correlation coefficient	0.2805	0.5993	−0.3592	0.4950	−0.4184

**FIGURE 3:**
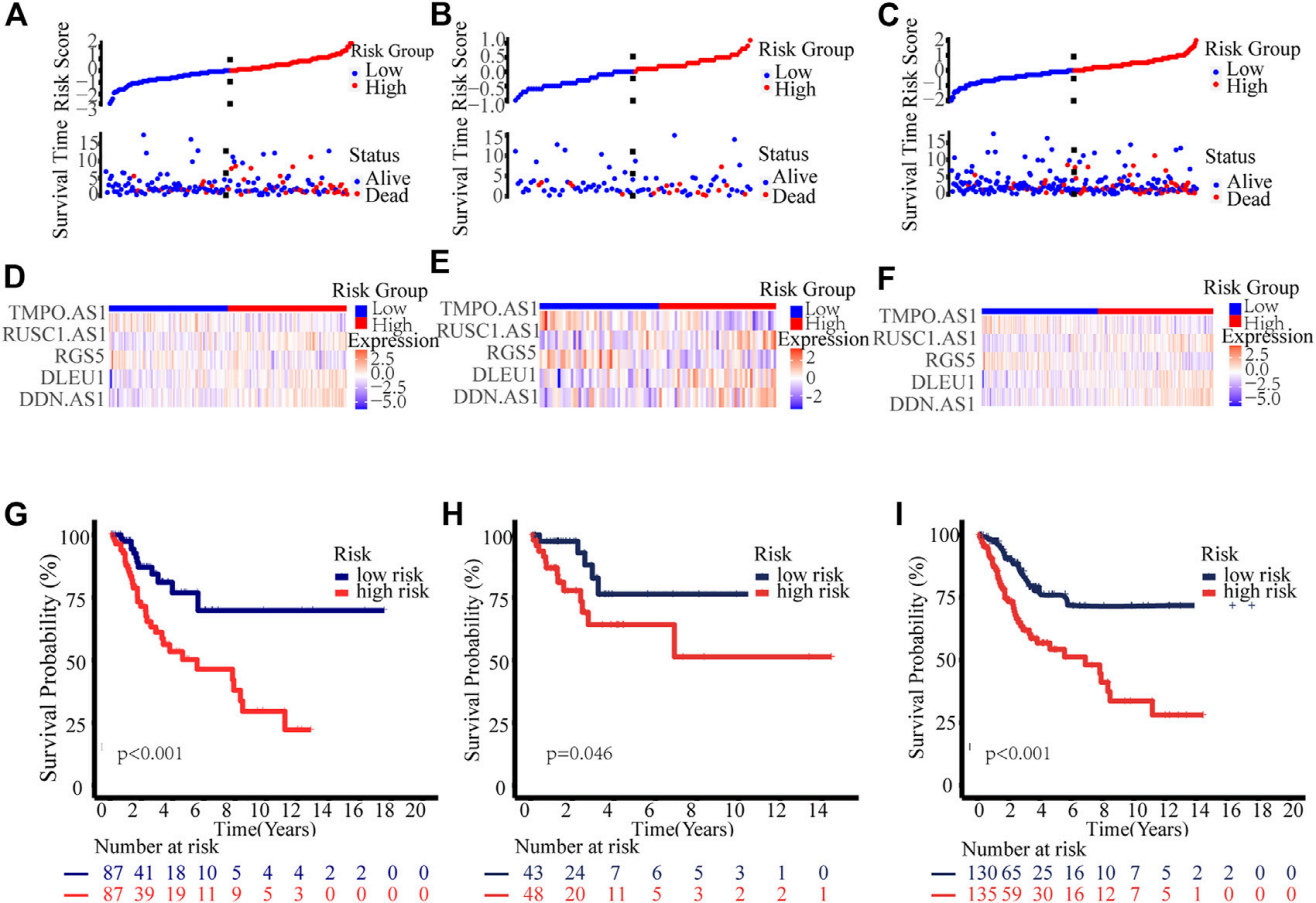
Prognosis value of the novel necroptosis-related lncRNAs prognostic model. Exhibition of the novel necroptosis-related lncRNAs signature based on risk score of the training, testing, and entire sets, respectively. Survival time and survival status between low and high-risk groups in the training **(A)**, testing **(B)**, and entire sets **(C)**, respectively. Heat map of five lncRNAs expression in the training **(D)**, test **(E)**, and entire sets **(F)**, respectively. Kaplan–Meier survival curves of OS of patients between low- and high-risk groups in the training **(G)**, test **(H)**, and entire sets **(I)**, respectively.

Thus, all cervical cancer patients were classified as low- or high-risk group according to the median risk score calculated by the above formula, As shown in Figure 3, patients in the high-risk group had significantly shorter survival times than those in the low-risk group, whether in the training group, test group or entire group. In addition, stratification analyses based on clinicopathologic characteristics, including age, gender, grade, stage, T, M, and N, showed that this signature was significantly associated with OS in all sub-groups (Supplementary Figure S3).

### 3.3 Construction of a nomogram based on necroptosis-related LncRNAs signature

**FIGURE 4:**
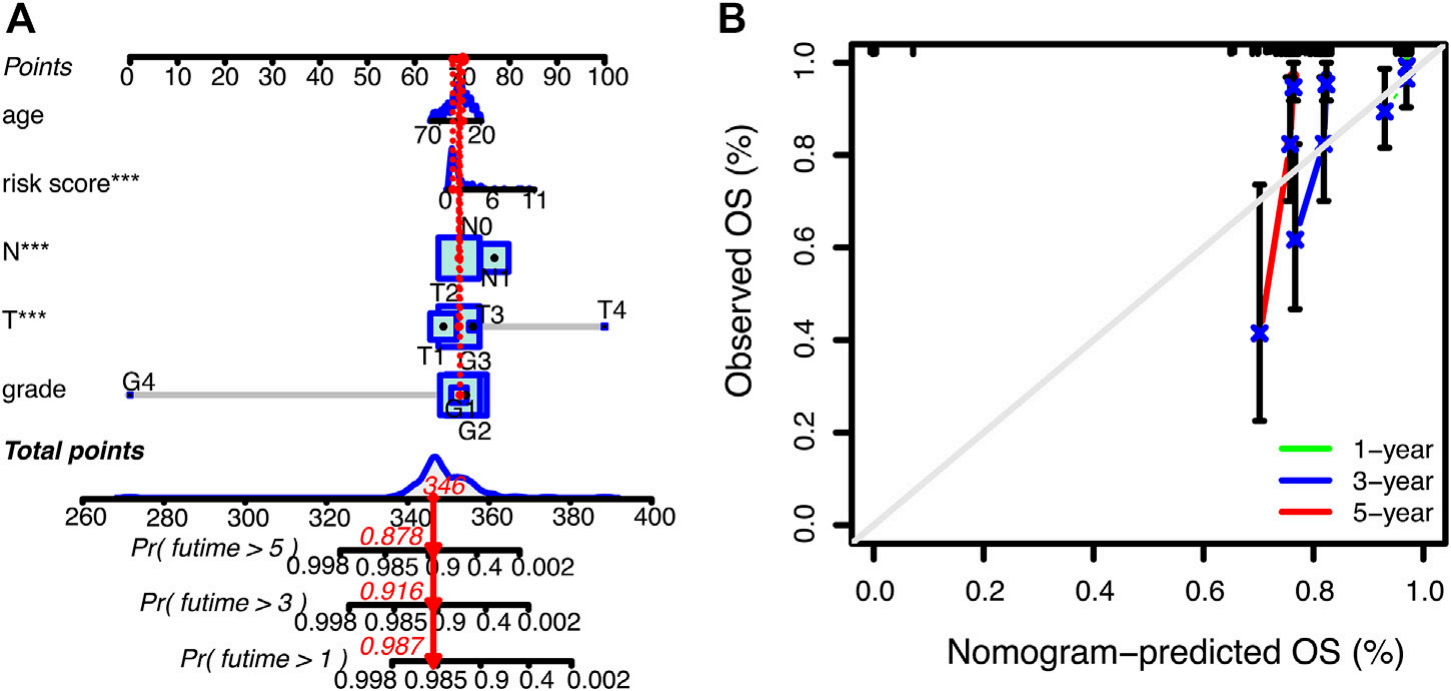
Nomogram for predicting overall survival (OS) in patients with cervical cancer. **(A)** Nomogram integrating signature, age, and tumor stage predicts the probability 1-year, 3-year, and 5-year OS. **(B)** Calibration curves for 1-year, 3-year, and 5-year OS.

Subsequently, we further explored the prognostic value of necrosis-associated lncRNAs signature. As shown in Table 2, the signature was confirmed as an independent prognostic predictor in univariate Cox regression (HR = 1.351, 95%CI = 1.161-1.573), and multivariate Cox regression (HR = 1.299, 95%CI = 1.119-1.508). In addition to the signature, T4 (101.837 and 14.825-699.563; *p* < 0.001) and N1 (3.942 and 1.799-8.636; *p* < 0.001) were identified as independent prognostic factors (Table 2). Next, we constructed a nomogram based on the three independent prognostic factors mentioned above: necroptosis-related lncRNAs signature, age, and TNM stage grade (Figure 4A). Using the 1-year, 3-year, and 5-year calibration plots, we found that the nomogram had a good concordance with the prediction of 1-year OS (Figure 4B).

**TABLE 2:** Univariate and multivariate Cox regression analysis of clinical factors and risk score with OS.

ID	Univariate analysis			Multivariate analysis		
	HR	95%CI	*p*-value	HR	95%CI	*p*-value
Risk score	1.351	1.161–1.573	<0.001	1.299	1.119–1.508	<0.001
Age	0.969	0.938–1.001	0.054	—	—	—
Grade-G2	1.315	0.626–2.766	0.470	—	—	—
Grade-G3	1.040	0.494–2.189	0.918	—	—	—
Grade-G4	<0.001	0∼∞	0.995	—	—	—
T-T2	0.550	0.190–1.588	0.269	0.482	0.160–1.451	0.194
T-T3	1.701	0.398–7.267	0.474	1.396	0.321–6.067	0.656
T-T4	267.841	36.009–1992.260	<0.001	101.837	14.825–699.563	<0.001
N-N1	3.902	1.842–8.268	<0.001	3.942	1.799–8.636	<0.001

**FIGURE 5:**
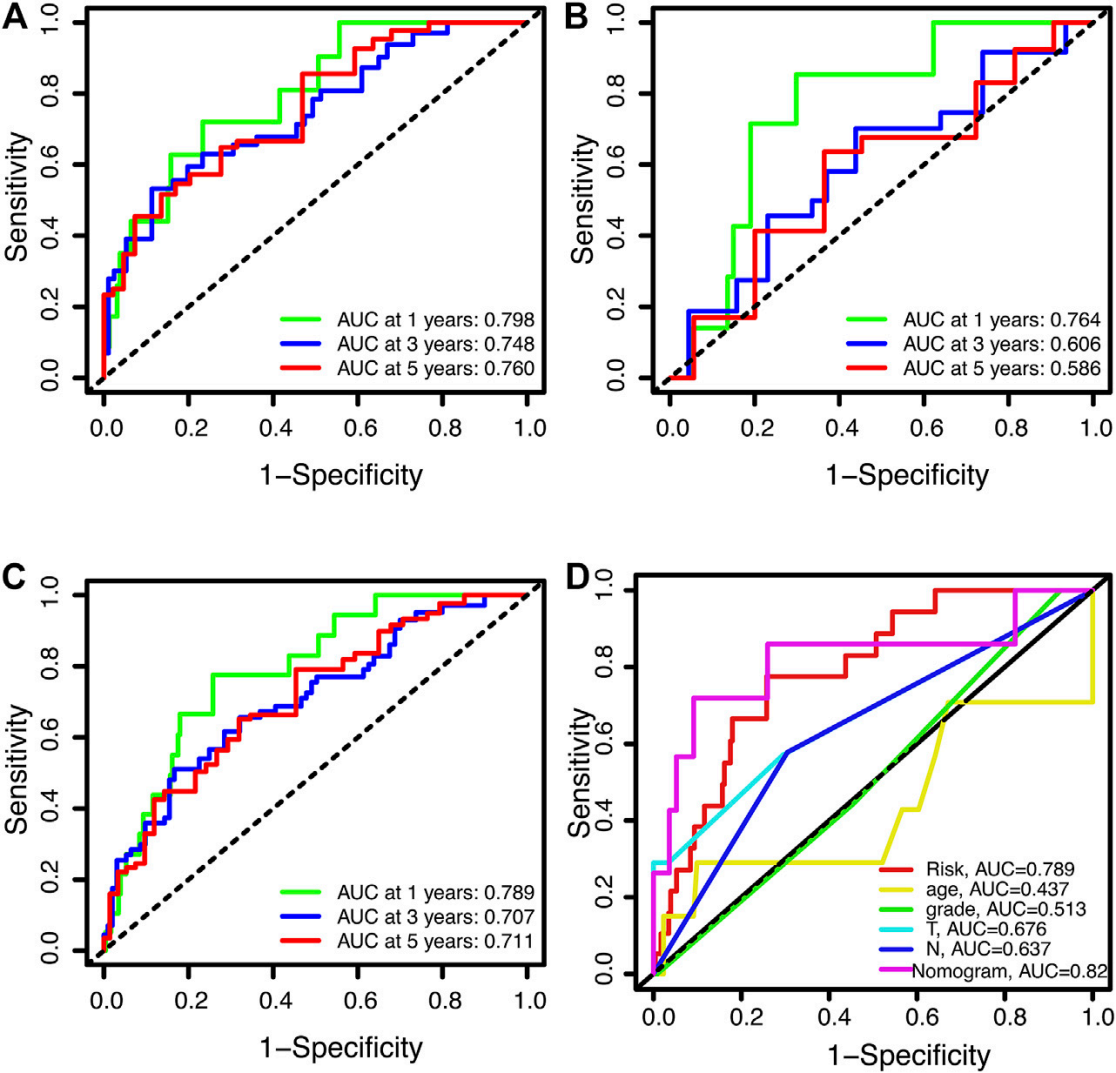
Receiver operating characteristic curves for overall survival (OS). **(A,B)** ROC curves for 1-year, 3-year, and 5-year OS in training **(A)**, testing **(B)**, and the entire sets **(C)**, respectively. **(D)** The 1-year OS ROC curves foe signature, clinical features and nomogram.

Time-dependent receiver operating characteristics (ROC) were used to evaluate the sensitivity and specificity of this novel signature on the prognosis. The area under curve (AUC) for the 1-year, 3-year, and 5-year OS of this signature were 0.798, 0.748, and 0.760 in the training set, 0.764, 0.606, and 0.586 in the test set, 0.789, 0.707, and 0.711 in the entire set, respectively (Figures 5A–C). The AUCs of the signature and the nomogram for 1-year OS were 0.789 and 0.821 respectively, which were superior to the clinical parameters (age, grade, T, N) (Figure 5D).

### 3.4 Altered pathways based on necroptosis-related LncRNAs signature

**FIGURE 6:**
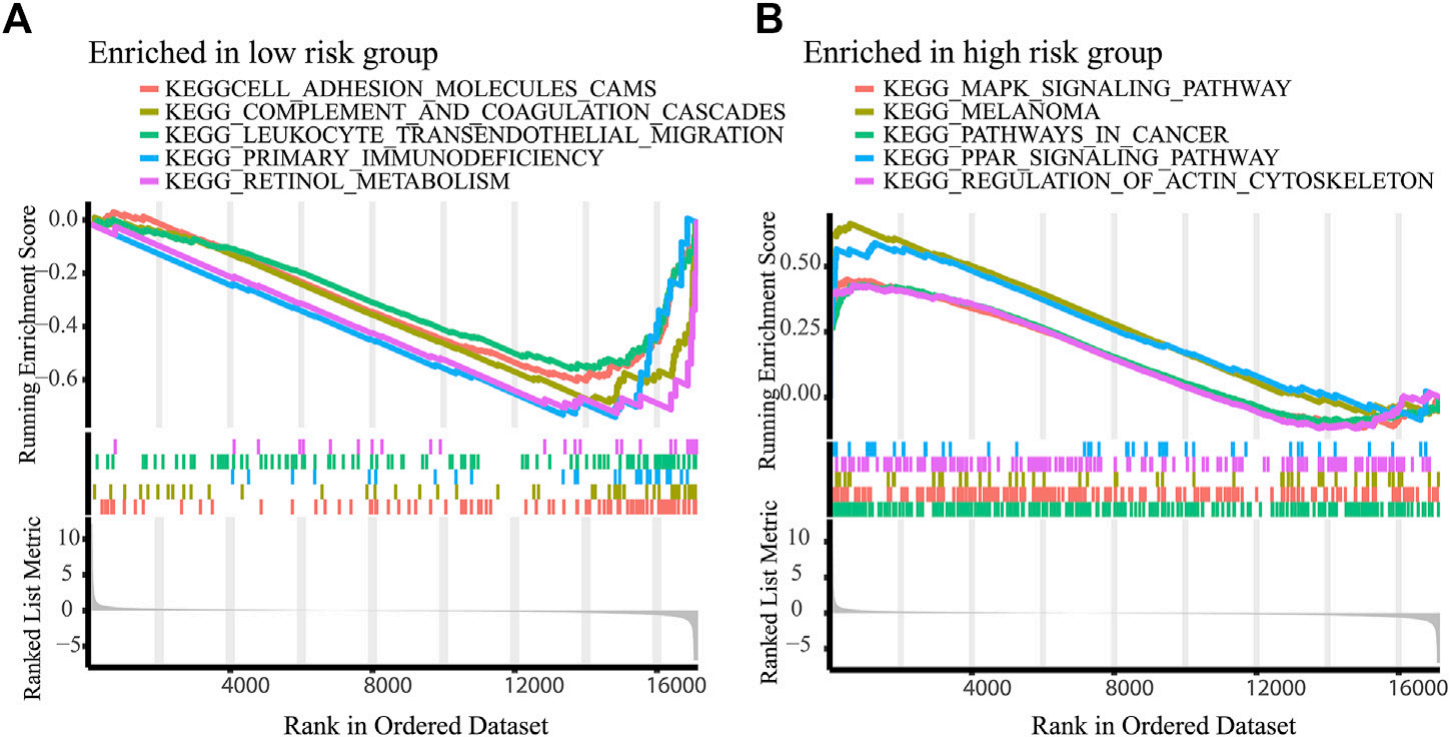
Activated pathways analyzed by GSEA. GSEA of the top 10 pathways significantly enriched in the low-**(A)** and high-risk group **(B)**.

To explore the possible signaling pathways involved in necroptosis-related lncRNAs, GSEA was performed. As shown in Figure 6, we found that leukocyte transendothelial migration, primary immunodeficiency, intestinal immune network for IgA production, complement and coagulation cascades were reached in the low-risk group, while retinol metabolism, PPAR signaling pathway, pathways in cancer, melanoma, MAPK signaling pathway were enriched in the high-risk group (*p* < 0.05). Notably, immune-related pathways were enriched in the low-risk group. Accordingly, we performed immunoassays on the established signature, especially in the low-risk group.

### 3.5 Immune-related analysis based on necroptosis-related LncRNAs signature

**FIGURE 7:**
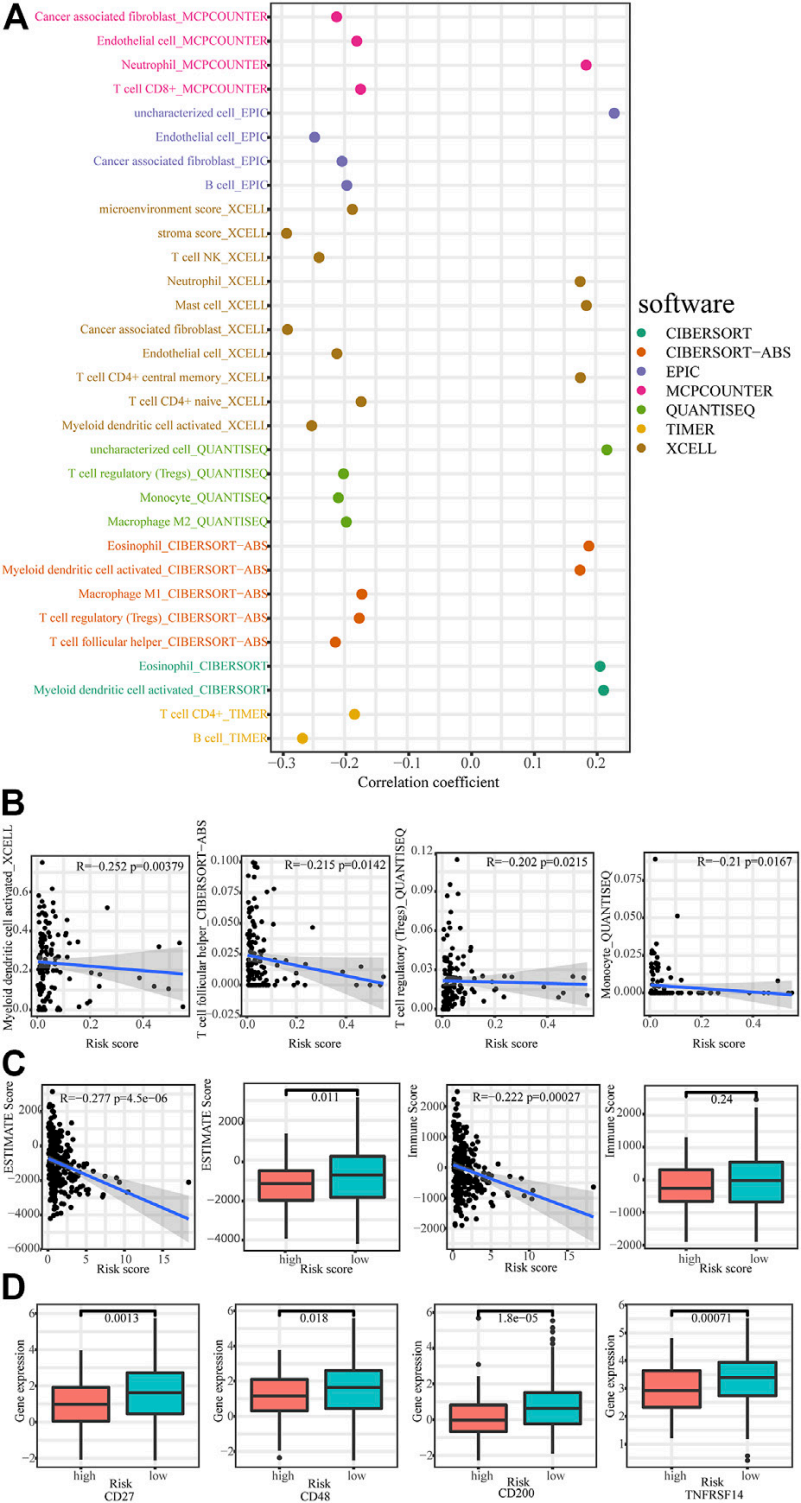
Immunoassays based on necroptosis-related lncRNAs signature. **(A,B)** The correlation between immune cells and risk scores. **(C)** Comparison of immune-related scores between low- and high-risk groups. **(D)** Comparison of checkpoints expression between low- and high-risk group.

Using TIMER, CIBERSORT, XCELL, QUANTISEQ, MCPcounter, EPIC, and CIBERSORT algorithm, we explored the correlation between immune cells and risk score in the low-risk group and found majority of immune cells were negatively correlated with risk score (Figures 7A,B). We also calculated the correlation between immune scores and signature. As show in Figure 7C, necroptosis-related lncRNAs signature showed a strong negative correlation with ESTIMAT scores and immune scores of cervical cancers. Moreover, ESTIMAT scores were significantly higher in the low-risk group than in the high-risk group. Moreover, immune checkpoint analysis showed higher expression of CD27, CD48, CD200, and TNFRSF14 in the low-risk group (Figure 7D).

### 3.6 Prediction of treatment response based on necroptosis-related LncRNAs signature

**FIGURE 8:**
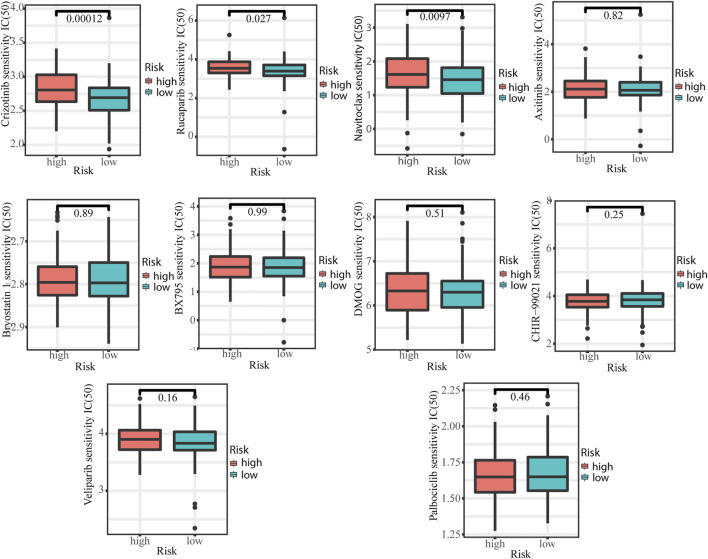
Comparison of IC50 of commonly targeting agents between low- and high-risk groups.

We explored the relationship between the signature and response to commonly targeted agents by calculating the IC50 and observed that patients in the low-risk group were more sensitive to Crizotinib (*p* = 0.00012), Rucaparib (*p* = 0.027), and Navitoclax (*p* = 0.0097) than those in high-risk group (Figure 8).

## 4 Discussion

To our knowledge, this study is the first analysis of necrosis-associated lncRNAs in cervical cancer and will help provide an important basis for future studies. Initially, we identified five necrosis-related lncRNAs, including lncRNA DDN-AS1, lncRNA DLEU1, lncRNA RGS5, lncRNA RUSC1-AS1, and lncRNA TMPO-AS1, for the construction of a signature that independently predict cervical cancer prognosis. So far, lncRNA DDN-AS1, lncRNA TMPO-AS1, lncRNA RUSC1-AS1, and lncRNA DLEU1 have been detected to be upregulated in cervical cancer and acted as promoters in cervical carcinogenesis (Liu et al., 2018; Liu et al., 2019; Gang et al., 2020; Guo et al., 2020). LncRNA DDN-AS1-miR-15a/16-TCF3 feedback loop contributed to proliferation, migration, and invasion in cervical cancer (Liu et al., 2019). LncRNA TMPO-AS1 promoted proliferation, migration, and invasion of cervical cancer cell by regulating the miR-143-3p/ZEB1 axis (Gang et al., 2020). LncRNA RUSC1-AS1 was reported to promote cervical cancer tumorigenesis by upregulating the output of miR-744-Bcl-2 axis (Guo et al., 2020). LncRNA DLEU1 promoted cervical cancer cell proliferation and invasion *via* the miR-381/HOXA13 axis. In addition, overexpression of DLEU1 (Liu et al., 2018) and RUSC1-AS1 (Guo et al., 2020) was significantly associated with shorter overall survival of patients with cervical cancer. Our study is consistent with these previous reporters and reveals an important role of necrosis-associated lncRNAs in promoting the growth and/or progression of cervical cancer. RGS5 (regulator of G protein signaling 5), a family of GTPase activating proteins, is extensively up-regulated in a variety of malignant cells, including non-small cell lung cancer (Huang et al., 2012), renal cell carcinoma (Furuya et al., 2004), and ovarian cancer (Wang et al., 2019), and is associated with tumor growth and poor prognosis. The RGS5-TGFβ-Smad2/3 axis converts pro-to anti-apoptotic signaling in tumor-residing pericytes and assists tumor growth (Dasgupta et al., 2021). RGS5 reduces proliferation of cancer cell derived primary endothelial cells *via* MAPK/ERK signaling pathway under hypoxia (Wang et al., 2019) in ovarian cancer. Our results suggested that RGS5 is up-regulated in cervical cancer, but its molecular biological function needs further investigation.

Recently, there is appreciable evidence that necroptosis-related lncRNAs can predict cancer prognosis and immunotherapy response. Zhao et al. (2021) reported that necroptosis-related lncRNAs could predict prognosis and help distinguish between cold and hot tumors in gastric cancer. In the study by Lu et al. (2022), a signature of seven necroptosis-related lncRNAs independently predicted the prognosis of patients with lung adenocarcinoma and provides guidance for immunotherapy. Similar results were obtained by the group of Liu et al. (2022), a signature of six necroptosis-related lncRNAs predicted survival in colon cancer and guided clinical drug. Our current study reported for the first time that a signature based on five necroptosis-related lncRNAs was an independent prognostic predictor of cervical cancer. Further ROC analysis confirmed that this signature predicted prognosis better than the clinical parameters (age, grade, T, N). Taken together, necroptosis-related lncRNAs may be clinically sensitive indicators for assessing the prognosis of patients with cervical cancer.

Necroptosis, a regulated cell death mainly mediated by RIPK1 (receptor-interacting protein [RIP] kinase 1), RIPK3, and MLKL (mixed lineage kinase domain-like pseudokinase) (Gong et al., 2019), has recently been reported to play significant roles in regulating cancer progression and is considered to be a trigger and amplifier of antitumor immunity in cancer therapy. In the present study, we detected several top altered pathways based on necrosis-associated lncRNA signatures that are immune-related, which further supports the notion that necrosis alters the tumor immune microenvironment.

Immune cells play a crucial role in antitumor activity, and many studies have used immune cells to exert antitumor effects, such as the destructive CAR-T cell (chimeric antigen receptor) therapies (Nair and Westin, 2020), and dendritic cell (DC) vaccines (Santos and Butterfield, 2018). Zhang et al. (2021) found that the main immune cell types in cervical cancer are B cells, T cells, natural killer cells (NK), and macrophages, and a recent study found that risk scores in an immune-related lncRNA based prognostic model of cervical cancer negatively correlated with macrophages M1, macrophages M2, myeloid dendritic cell, and CD8 + T cells, and positively correlated with macrophages M0 (Lv et al., 2022). In our study, we found that the risk score for necroptosis-related lncRNA prognostic signature was negatively correlated with most immune cells, including monocyte, myeloid dendritic cell activated, T cell follicular helper, T cell regulatory (Tregs) in the low-risk group. In summary, based on the enrichment of immune-related pathways in the low-risk group and the negative correlation of risk scores with most immune cells, we hypothesized that patients in the low-risk group might be more suitable for immunotherapy and our model could be used to assess the efficacy and prognosis of immunotherapy for patients.

Rucaparib is a PAPP inhibitor that has shown potential value as a novel targeted agent in the treatment of cervical cancer. Rucaparib exerts significant anti-proliferative effects and acts as an effective radiosensitizer in cervical cancer (Tang et al., 2019). Rucaparib antagonizes multidrug resistance in cervical cancer cells by blocking the function of ABC transporters (Chen et al., 2020). Our current study revealed that cervical cancer patients in the low-risk group exhibiting greater sensitivity to Rucaparib, suggesting that a novel signature based on necroptosis-related lncRNAs is a predictor of Rucaparib efficacy. The underlying mechanism may be that PARP-1 expression/activity may be upregulated in response to ongoing oxidative stress (HPV and inflammation) and further promote progression through NF-JB and NAD + depletion-induced necrosis (Tomao et al., 2020). Crizotinib, an inhibitor of mesenchymal epithelial transforming factor (c-MET) and anaplastic lymphoma kinase (ALK), has shown promising anticancer effects in cervical cancer. Crizotinib induces anticancer activity in human cervical cancer cells through ROS-dependent mitochondrial depolarization and induction of apoptosis pathways (Varma and Tiwari, 2021). In addition, the sensitivity of crizotinib could be enhanced by Na+/H+ exchanger regulator factor 1 (NHERFI) (Yang et al., 2020). Our current study also showed cervical cancer patients in low-risk groups were more sensitivity to crizotinib. Collectively, our findings suggest that the low-risk patients may be more suitable for treatment with rucaparib and crizotinib, and our findings will help accelerate the exploration of rucaparib and crizotinib for the treatment of cervical cancer. Navitoclax is a novel inhibitor of Bcl-2 family proteins and has shown encouraging efficacy in small-cell lung cancer (Gandhi et al., 2011). There are few studies on Navitoclax in cervical cancer. In the current study, we showed that cervical cancer patients in low-risk groups were more sensitivity to Navitoclax, however, further studies are needed to determine whether Navitoclax can be used in the treatment of cervical cancer.

In the current study, we first constructed a prognostic model for necrosis-associated lncRNAs using univariate and multivariate Cox regression analysis combined with LASSO regression to improve the accuracy and validity of the predictive model. Then, an easy-to-use line graph model was constructed based on Cox analysis in carefully screened patients with clinicopathological features, which would facilitate prediction in individual patients. We further evaluated the prognostic signature using ROC curves and AUC and confirmed that the signature showed good predictive value. Although we used many methods to successfully optimize our model, there are still some drawbacks and deficiencies. Firstly, this study is a retrospective study and there is some bias in the data. Second, we have performed internal validation with the test set and entire set in the model, but it is difficult to externally validate the prognosis. Finally, the mechanism by which these lncRNAs affect necroptosis remains unknown. Further functional and mechanistic studies of necroptosis-related lncRNAs are necessary.

## 5 Conclusion

In conclusion, we established a signature based on five necroptosis-related lncRNA that can independently predict cervical cancer prognosis. In addition, this signature can predict treatment response in cervical cancer. We found patients with cervical cancer in the low-risk group showed more sensitivity to rucaparib, crizotinib, and navitoclax, and our findings will help accelerate the exploration of these drugs for the treatment of cervical cancer.

## Data Availability

The original contributions presented in the study are included in the article/Supplementary Material, further inquiries can be directed to the corresponding author.

## References

[B1] Burk R. D. Chen Z. Saller C. Tarvin K. Carvalho A. L. Scapulatempo-Neto C. (2017). Integrated genomic and molecular characterization of cervical cancer. Nature 543 (7645), 378–384. 10.1038/nature21386 28112728 PMC5354998

[B2] Chen F. Yang J. Fang M. Wu Y. Su D. Sheng Y. (2022). Necroptosis-related lncRNA to establish novel prognostic signature and predict the immunotherapy response in breast cancer. J. Clin. Lab. Anal. 36, 4. 10.1002/jcla.24302 PMC8993638 35229919

[B3] Chen Z. Ling K. Zhu Y. Deng L. Li Y. Liang Z. (2020). Rucaparib antagonize multidrug resistance in cervical cancer cells through blocking the function of ABC transporters. Gene 759, 145000. 10.1016/j.gene.2020.145000 32717310

[B4] Cohen P. A. Jhingran A. Oaknin A. Denny L. (2019). Cervical cancer. Lancet 393, 169–182. 10.1016/S0140-6736(18)32470-X 30638582

[B5] Dasgupta S. Ghosh T. Dhar J. Bhuniya A. Nandi P. Das A. (2021). RGS5-TGFβ-Smad2/3 axis switches pro- to anti-apoptotic signaling in tumor-residing pericytes, assisting tumor growth. Cell Death Differ. 28 (11), 3052–3076. 10.1038/s41418-021-00801-3 34012071 PMC8564526

[B6] Furuya M. Nishiyama M. Kimura S. Suyama T. Naya Y. Ito H. (2004). Expression of regulator of G protein signalling protein 5 (RGS5) in the tumour vasculature of human renal cell carcinoma. J. Pathol. 203 (1), 551–558. 10.1002/path.1543 15095478

[B7] Gandhi L. Camidge D. R. Ribeiro de Oliveira M. Bonomi P. Gandara D. Khaira D. (2011). Phase I study of Navitoclax (ABT-263), a novel Bcl-2 family inhibitor, in patients with small-cell lung cancer and other solid tumors. J. Clin. Oncol. 29 (7), 909–916. 10.1200/JCO.2010.31.6208 21282543 PMC4668282

[B8] Gang X. Yuan M. Zhang J. (2020). Long non-coding RNA TMPO-AS1 promotes cervical cancer cell proliferation, migration, and invasion by regulating miR-143-3p/ZEB1 Axis. Cancer Manag. Res. 12, 1587–1599. 10.2147/cmar.S226409 32184662 PMC7060785

[B9] Gao Y. Zou T. Liang W. Zhang Z. Qie M. (2021). Long non-coding RNA HAND2-AS1 delays cervical cancer progression via its regulation on the microRNA-21-5p/TIMP3/VEGFA axis. Cancer Gene Ther. 28 (6), 619–633. 10.1038/s41417-020-00243-y 33139818

[B10] Ghosh D. Khajanchi S. Mangiarotti S. Denis F. Dana S. K. Letellier C. (2017). How tumor growth can be influenced by delayed interactions between cancer cells and the microenvironment? Biosystems. 158, 17–30. 10.1016/j.biosystems.2017.05.001 28506827

[B11] Gong Y. Fan Z. Luo G. Yang C. Huang Q. Fan K. (2019). The role of necroptosis in cancer biology and therapy. Mol. Cancer 18, 100. 10.1186/s12943-019-1029-8 31122251 PMC6532150

[B12] Guo Q. Zhang Q. Lu L. Xu Y. (2020). Long noncoding RNA RUSC1-AS1 promotes tumorigenesis in cervical cancer by acting as a competing endogenous RNA of microRNA-744 and consequently increasing Bcl-2 expression. Cell Cycle 19 (10), 1222–1235. 10.1080/15384101.2020.1749468 32264732 PMC7217379

[B13] Harari-Steinfeld R. Gefen M. Simerzin A. Zorde-Khvalevsky E. Rivkin M. Ella E. (2021). The lncRNA H19-derived MicroRNA-675 promotes liver necroptosis by targeting FADD. Cancers (Basel) 13, 411. 10.3390/cancers13030411 33499244 PMC7866230

[B14] Huang G. Song H. Wang R. Han X. Chen L. (2012). The relationship between RGS5 expression and cancer differentiation and metastasis in non-small cell lung cancer. J. Surg. Oncol. 105 (4), 420–424. 10.1002/jso.22033 21780128

[B15] Huarte M. (2015). The emerging role of lncRNAs in cancer. Nat. Med. 21 (11), 1253–1261. 10.1038/nm.3981 26540387

[B16] Jiang N. Zhang X. Gu X. Li X. Shang L. (2021). Progress in understanding the role of lncRNA in programmed cell death. Cell Death Discov. 7, 30. 10.1038/s41420-021-00407-1 33558499 PMC7870930

[B17] Khajanchi S. Nieto J. J. (2021). Spatiotemporal dynamics of a glioma immune interaction model. Sci. Rep. 11 (1), 22385. 10.1038/s41598-021-00985-1 34789751 PMC8599515

[B18] Li L. Yu S. Zang C. (2018). Low necroptosis process predicts poor treatment outcome of human papillomavirus positive cervical cancers by decreasing tumor-associated macrophages M1 polarization. Gynecol. Obstet. Invest. 83 (3), 259–267. 10.1159/000487434 29621771

[B19] Liu C. Tian X. Zhang J. Jiang L. (2018). Long non-coding RNA DLEU1 promotes proliferation and invasion by interacting with miR-381 and enhancing HOXA13 expression in cervical cancer. Front. Genet. 9, 629. 10.3389/fgene.2018.00629 30581456 PMC6292861

[B20] Liu L. Huang L. Chen W. Zhang G. Li Y. Wu Y. (2022). Comprehensive analysis of necroptosis-related long noncoding RNA immune infiltration and prediction of prognosis in patients with colon cancer. Front. Mol. Biosci. 9, 811269. 10.3389/fmolb.2022.811269 35237659 PMC8883231

[B21] Liu Z. Wu M. Shi H. Huang C. Luo S. Song X. (2019). DDN-AS1-miR-15a/16-TCF3 feedback loop regulates tumor progression in cervical cancer. J. Cell. Biochem. 120 (6), 10228–10238. 10.1002/jcb.28307 30582201

[B22] Lu Y. Luo X. Wang Q. Chen J. Zhang X. Li Y. (2022). A novel necroptosis-related lncRNA signature predicts the prognosis of lung adenocarcinoma. Front. Genet. 13, 862741. 10.3389/fgene.2022.862741 35368663 PMC8969905

[B23] Luo L. Li L. Liu L. Feng Z. Zeng Q. Shu X. (2022). A necroptosis-related lncRNA-based signature to predict prognosis and probe molecular characteristics of stomach adenocarcinoma. Front. Genet. 13, 833928. 10.3389/fgene.2022.833928 35330731 PMC8940523

[B24] Lv X. Liu L. Li P. Yuan Y. Peng M. Jin H. (2022). Constructing a novel signature based on immune-related lncRNA to improve prognosis prediction of cervical squamous cell carcinoma patients. Reprod. Sci. 29 (3), 800–815. 10.1007/s43032-022-00851-z 35075611

[B25] Mohanty S. Yadav P. Lakshminarayanan H. Sharma P. Vivekanandhan A. Karunagaran D. (2022). RETRA induces necroptosis in cervical cancer cells through RIPK1, RIPK3, MLKL and increased ROS production. Eur. J. Pharmacol. 920, 174840. 10.1016/j.ejphar.2022.174840 35219733

[B26] Nair R. Westin J. (2020). CAR T-Cells. Adv. Exp. Med. Biol. 1244, 215–233. 10.1007/978-3-030-41008-7_10 32301017

[B27] Qin D. Lv X. Liu L. Li P. Yuan Y. Peng M. (2021). Constructing A novel signature based on immune-related lncrna to improve prognosis prediction of cervical squamous cell carcinoma patients. Reprod. Sci. 29, 800–815. 10.21203/rs.3.rs-640056/v1 35075611

[B28] Santos P. M. Butterfield L. H. (2018). Dendritic cell-based cancer vaccines. J. Immunol. 200, 443–449. 10.4049/jimmunol.1701024 29311386 PMC5880540

[B29] Sardar M. Khajanchi S. Biswas S. Abdelwahab S. F. Nisar K. S. (2021). Exploring the dynamics of a tumor-immune interplay with time delay. Alexandria Eng. J. 60 (5), 4875–4888. 10.1016/j.aej.2021.03.041

[B30] Schmidt S. V. Seibert S. Walch-Rückheim B. Vicinus B. Kamionka E. M. Pahne-Zeppenfeld J. (2015). RIPK3 expression in cervical cancer cells is required for PolyIC-induced necroptosis, IL-1α release, and efficient paracrine dendritic cell activation. Oncotarget 6 (11), 8635–8647. 10.18632/oncotarget.3249 25888634 PMC4496172

[B31] Sung H. Ferlay J. Siegel R. L. Laversanne M. Soerjomataram I. Jemal A. (2021). Global cancer statistics 2020: GLOBOCAN estimates of incidence and mortality worldwide for 36 cancers in 185 countries. Ca. Cancer J. Clin. 71 (3), 209–249. 10.3322/caac.21660 33538338

[B32] Tang M. Liu Q. Zhou L. Chen L. Yang X. Yu J. (2019). The poly (ADP-ribose) polymerase inhibitor rucaparib suppresses proliferation and serves as an effective radiosensitizer in cervical cancer. Invest. New Drugs 37 (1), 65–75. 10.1007/s10637-018-0616-7 29872938

[B33] Tomao F. Santangelo G. Musacchio L. Di Donato V. Fischetti M. Giancotti A. (2020). Targeting cervical cancer: Is there a role for poly (ADP-ribose) polymerase inhibition? J. Cell. Physiol. 235 (6), 5050–5058. 10.1002/jcp.29440 31912897

[B34] Varma D. A. Tiwari M. (2021). Crizotinib-induced anti-cancer activity in human cervical carcinoma cells via ROS-dependent mitochondrial depolarization and induction of apoptotic pathway. J. Obstet. Gynaecol. Res. 47 (11), 3923–3930. 10.1111/jog.15003 34482598

[B35] Wang D. Xu Y. Feng L. Yin P. Song S. S. Wu F. (2019). RGS5 decreases the proliferation of human ovarian carcinoma-derived primary endothelial cells through the MAPK/ERK signaling pathway in hypoxia. Oncol. Rep. 41 (1), 165–177. 10.3892/or.2018.6811 30365142 PMC6278583

[B36] Wang K. Liu F. Liu C. Y. An T. Zhang J. Zhou L. Y. (2016). The long noncoding RNA NRF regulates programmed necrosis and myocardial injury during ischemia and reperfusion by targeting miR-873. Cell Death Differ. 23 (8), 1394–1405. 10.1038/cdd.2016.28 27258785 PMC4947670

[B37] Wang Y. Farmer M. Izaguirre E. W. Schwartz D. L. Somer B. Tillmanns T. (2018). Association of definitive pelvic radiation therapy with survival among patients with newly diagnosed metastatic cervical cancer. JAMA Oncol. 4 (9), 1288–1291. 10.1001/jamaoncol.2018.2677 30054609 PMC6143011

[B38] Xu Y. Zhu H. Ma H. Yuan L. Hu Q. Yang L. (2020). LINC01305 inhibits malignant progression of cervical cancer via miR-129-5p/Sox4 axis. Am. J. Transl. Res. 12 (11), 7581–7592. 33312390 PMC7724335

[B39] Yan J. Wan P. Choksi S. Liu Z. G. (2022). Necroptosis and tumor progression. Trends Cancer 8 (1), 21–27. 10.1016/j.trecan.2021.09.003 34627742 PMC8702466

[B40] Yang F. Hu M. Chang S. Huang J. Si Y. Wang J. (2020). Alteration in the sensitivity to crizotinib by Na(+)/H(+) exchanger regulatory factor 1 is dependent to its subcellular localization in ALK-positive lung cancers. BMC Cancer 20 (1), 202. 10.1186/s12885-020-6687-9 32164629 PMC7068933

[B41] Zhang Y. Yu M. Jing Y. Cheng J. Zhang C. Cheng L. (2021). Baseline immunity and impact of chemotherapy on immune microenvironment in cervical cancer. Br. J. Cancer 124 (2), 414–424. 10.1038/s41416-020-01123-w 33087896 PMC7852680

[B42] Zhao Z. Liu H. Zhou X. Fang D. Ou X. Ye J. (2021). Necroptosis-related lncRNAs: Predicting prognosis and the distinction between the cold and hot tumors in gastric cancer. J. Oncol. 2021, 6718443. 10.1155/2021/6718443 34790235 PMC8592775

