# Supplementary material for: A novel necroptosis-related lncRNA based signature predicts prognosis and response to treatment in cervical cancer
Source: Front Genet. 2022 Dec 6;13:938250. doi: 10.3389/fgene.2022.938250 (PMC9763697; doi:10.3389/fgene.2022.938250)
Supplement: Supplementary file 1 [file DataSheet1.PDF]

Supplementary Material

Supplementary Table 1 | The coefficients (coef) of the 9 necroptosis-related lncRNAs detected by lasso regression analysis

LncRNAs	Coefficient
C1orf147	0.12956018079685
DDN-AS1	0.219760496574761
DLEU1	0.374738661909415
LINC02207	-0.0873393154464783
PRR7-AS1	0.122923518431497
RGS5	-0.21355650357555
RUSC1-AS1	0.180913914936472
TMPO-AS1	-0.302905512113855
UNQ6494	-0.0847592080630228

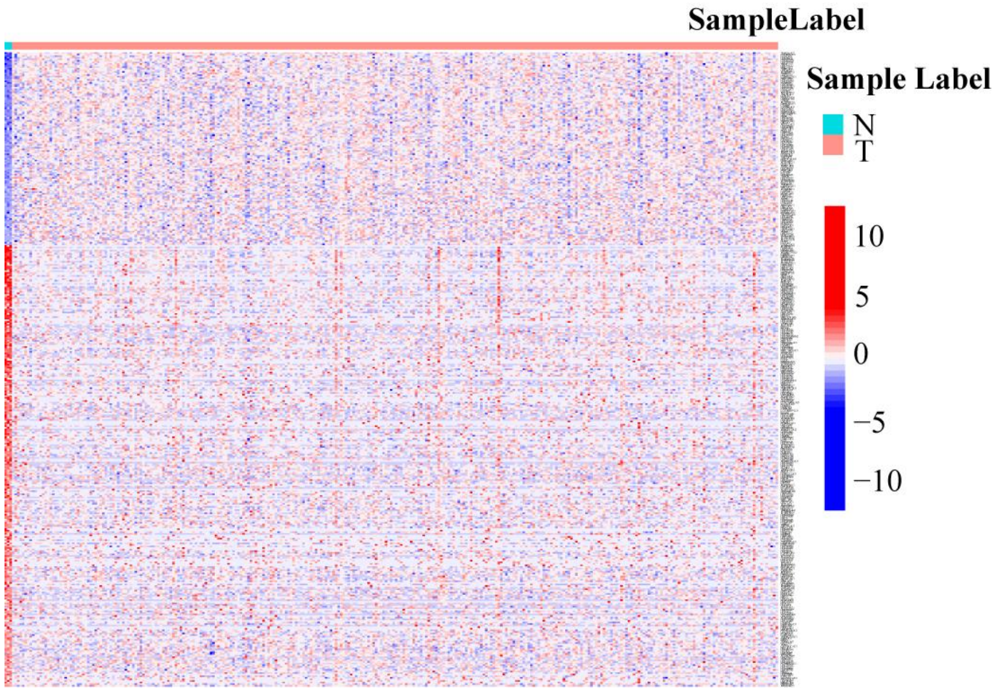

Supplementary Figure 1. Heatmap of differentially expressed lncRNAs in cervical cancer from TCGA dataset. Red and blue indicate up-regulated and down-regulated lncRNAs, respectively.

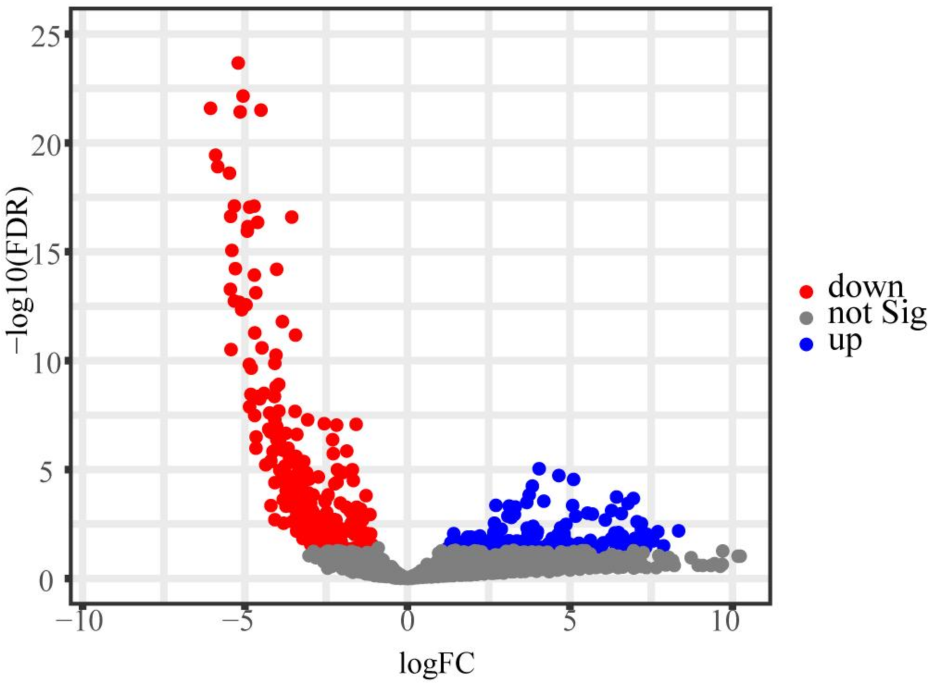

Supplementary Figure 2. Volcano plot of 119 differentially expressed necroptosis -related LncRNAs. Red and blue indicate up-regulated and down-regulated lncRNAs respectively.

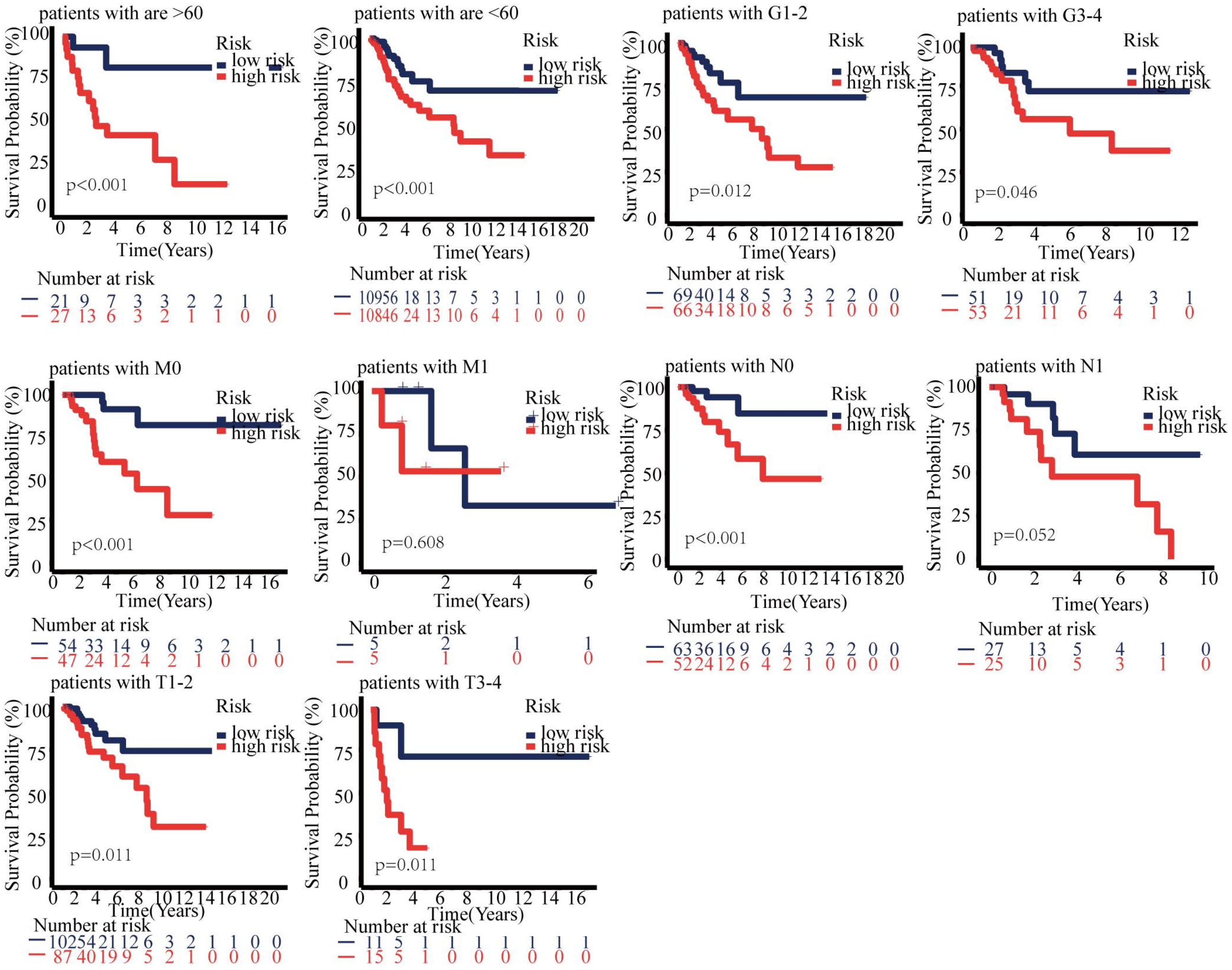

Supplementary Figure 3. Kaplan–Meier survival curves of overall survival stratified by age, gender, grade, stage, T, N, or M between low- and high-risk groups in the entire set.
